# Anesthetic Management for Carotid Endarterectomy Using Superficial and Intermediate Cervical Plexus Block: A Case Report

**DOI:** 10.7759/cureus.109662

**Published:** 2026-05-26

**Authors:** Roshini Thangaraj, Venkatesh Selvaraj

**Affiliations:** 1 Anesthesiology and Critical Care, Sri Ramachandra Institute of Higher Education and Research, Chennai, IND

**Keywords:** carotid endarterectomy, general anesthesia, intermediate cervical plexus block, regional anesthesia, superficial cervical plexus block

## Abstract

Carotid endarterectomy (CEA) is a surgical procedure that is performed to reduce the risk of stroke by removing atherosclerotic plaque from the carotid artery. Regional anesthesia, mainly a combined superficial and intermediate cervical plexus block, is used as an alternative to general anesthesia for maintaining cerebral perfusion, hemodynamic control, cerebral monitoring, and rapid neurological assessment during surgery. Here, we present a case of a 69-year-old male with symptomatic right carotid artery stenosis who underwent a successful right CEA using a combined superficial and intermediate cervical plexus block.

## Introduction

Carotid endarterectomy (CEA) is the gold standard surgical intervention for high-grade carotid artery stenosis [1]. Perioperative anesthetic management plays an important role in optimizing cerebral perfusion, maintaining hemodynamic stability, and reducing neurological complications during the procedure. While general anesthesia is commonly used, regional anesthesia offers advantages, including hemodynamic stability, reduced need for intensive postoperative care and prolonged elective ventilatory support, and the ability to assess neurologic status intraoperatively [2,3].

Regional anesthesia, commonly performed using a combined superficial and intermediate cervical plexus block, allows continuous assessment of the patient’s neurological status during carotid cross-clamping. It also facilitates early detection of cerebral ischemia and may reduce the need for shunt placement, minimize hemodynamic instability, and reduce postoperative complications. Successful implementation of this method requires careful patient selection, accurate block technique, and careful intraoperative monitoring. This report describes a case in which combined superficial and intermediate cervical plexus blocks were used effectively to manage a patient with recurrent cerebrovascular accident and comorbidities undergoing right-sided CEA.

## Case presentation

A 69-year-old male patient presented with chief complaints of left-sided upper and lower limb weakness (power of 2/5 in the left upper and lower limbs), with no history of trauma, loss of consciousness, headache, or seizure. The patient had a recurrent history of cerebrovascular accident (2019) and was on dual antiplatelets and statins (stopped prior to surgery), along with known hypertension and diabetes mellitus on treatment. The functional status of the patient was found to be less than 4 METs.

**Figure 1:**
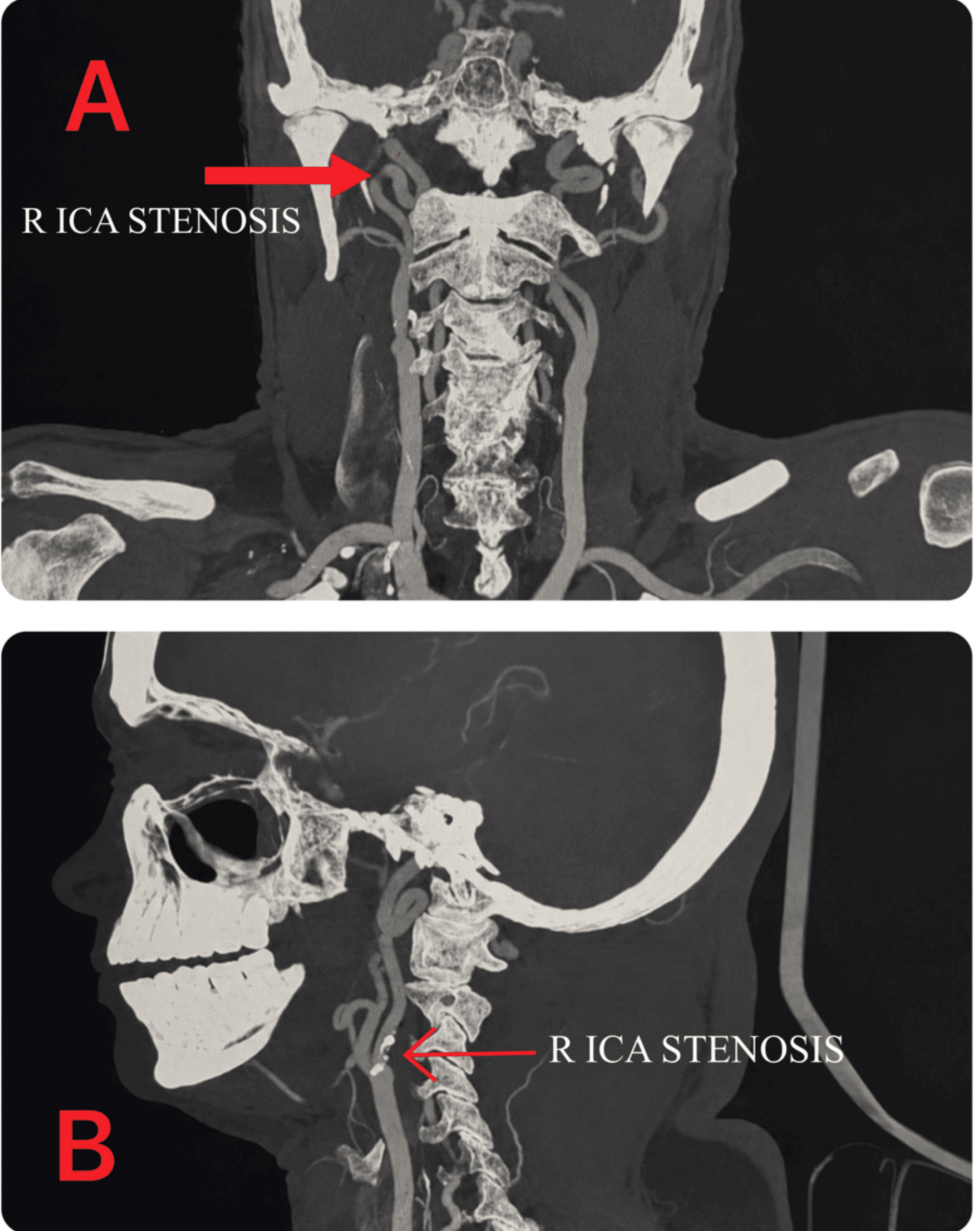
CT angiogram of neck vessels R ICA, right internal carotid artery

CT angiogram of the neck and intracranial vessels showed an eccentric atheromatous plaque causing 70-80% stenosis in the cervical segment of the right internal carotid artery (Figure 1).

Anesthetic management

After obtaining informed consent, a combined superficial and intermediate cervical plexus block was performed under ultrasound guidance. The patient was placed in the supine position with neck extension and head tilted toward the left side. All standard ASA monitors were applied. An invasive arterial line using a 20G Jelco catheter was placed in the left radial artery for invasive blood pressure monitoring. Rapid neurological assessment was performed using hand grip. Fentanyl 1 mcg/kg IV was administered for patient comfort while maintaining spontaneous ventilation.

Superficial and intermediate cervical plexus block

**Figure 2:**
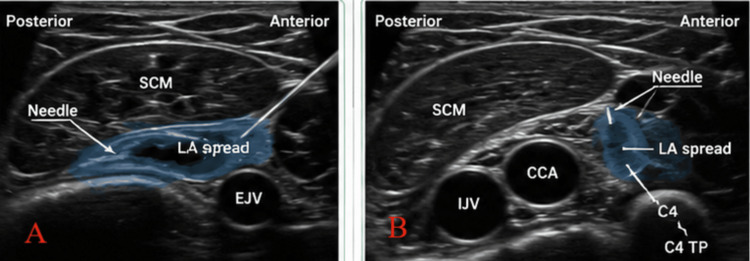
Ultrasound images of both superficial (A) and intermediate (B) cervical plexus block C4 TP, C4 transverse process; CCA, common carotid artery; EJV, external jugular vein; IJV, internal jugular vein; LA, local anesthetic; SCM, sternocleidomastoid

Under sterile aseptic precautions (Figure 2), using ultrasound guidance and a 50 mm Stimuplex needle, a combined superficial and intermediate cervical plexus block was performed using 25 mL of local anesthetic agent (7 mL of 0.5% bupivacaine and 7 mL of 2% lidocaine with adrenaline).

Surgical course

Within 10 minutes, adequate anesthesia was confirmed by sensory loss in C2-C4 dermatomes. The patient remained hemodynamically stable with a baseline blood pressure of 139/70 mmHg and responsive throughout the duration of the procedure (two hours). Neurological status was monitored intraoperatively through continuous verbal interaction and hand grip movements. Shunting of the internal carotid artery was not required. The patient was transferred to the postoperative care area fully awake with stable vitals and no neurological deficits. Analgesia was maintained with low-dose opioids and NSAIDs as needed.

## Discussion

Carotid endarterectomy is the gold standard surgical intervention for the prevention of recurrent ischemic stroke in patients with significant carotid artery stenosis [4]. The choice of anesthetic technique remains debated between general anesthesia and regional anesthesia [5,6]. However, in regional anesthesia, particularly cervical plexus blocks, several perioperative advantages are increasingly relevant in high-risk vascular patients.

Regional vs. general anesthesia

The combined superficial and intermediate cervical plexus block provides effective anesthesia for CEA while preserving spontaneous ventilation and consciousness, thereby enabling continuous neurological assessment during carotid cross-clamping [7]. This ability to directly monitor cerebral function is a major advantage over general anesthesia, where monitoring modalities such as electroencephalography, somatosensory evoked potentials, or near-infrared spectroscopy are required. Awake neurological monitoring allows early detection of cerebral hypoperfusion, facilitating timely shunt placement and potentially reducing perioperative stroke risk.

Superficial and intermediate cervical plexus block vs. deep cervical block

The superficial cervical plexus supplies cutaneous innervation to the anterolateral neck, while the intermediate cervical plexus, located superficial to the prevertebral fascia, provides anesthesia to deeper structures involved in carotid surgery [8,9]. Combining these two blocks enhances the extent of anesthesia without the increased risk associated with deep cervical plexus block, such as vertebral artery puncture or phrenic nerve paralysis. This makes the combined approach particularly suitable for elderly patients and those with compromised cardiopulmonary reserve.

Hemodynamic considerations

Hemodynamic stability is another important consideration in CEA. Regional anesthesia has been associated with reduced intraoperative blood pressure variability compared with general anesthesia [10]. During carotid cross-clamping, permissive hypertension can be more precisely titrated in an awake patient. Preservation of baroreceptor reflexes under regional anesthesia may reduce the incidence of significant perioperative hypotension [11-13].

Anesthetic agent considerations

Sedation under cervical plexus block must be carefully titrated to maintain patient cooperation and airway patency. Agents such as dexmedetomidine are favored due to their anxiolytic and analgesic properties with minimal respiratory depression.

## Conclusions

CEA can be effectively and safely performed under a combined superficial and intermediate cervical plexus block. Regional anesthesia not only provides the advantage of continuous neurological assessment during surgery but also maintains hemodynamic stability and may reduce perioperative complications associated with general anesthesia. This case highlights that the combined use of superficial and intermediate cervical plexus blocks may represent a feasible and effective anesthetic approach for selected patients undergoing CEA.
